# Establishing and validating an ADCP-related prognostic signature in pancreatic ductal adenocarcinoma

**DOI:** 10.18632/aging.204221

**Published:** 2022-08-12

**Authors:** Deyu Zhang, Fang Cui, Lisi Peng, Meiqi Wang, Xiaoli Yang, Chuanchao Xia, Keliang Li, Hua Yin, Yang Zhang, Qihong Yu, Zhendong Jin, Haojie Huang

**Affiliations:** 1 Department of Gastroenterology, Changhai Hospital, Shanghai, China; 2 Department of Gastroenterology, The First Affiliated Hospital of Zhengzhou University, China

**Keywords:** antibody-dependent cell phagocytosis, pancreatic ductal adenocarcinoma, gene signature

## Abstract

With the progress of precision medicine treatment in pancreatic ductal adenocarcinoma (PDAC), individualized cancer-related examination and prediction is of great importance in this high malignant tumor, and antibody-dependent cell phagocytosis (ADCP) with changed pathways highly enrolled in the carcinogenesis of PDAC. High-throughput data of pancreatic ductal adenocarcinoma were downloaded and 160 differentially expressed ADCP-related genes (ARGs) were obtained. Secondly, GO and KEGG enrichment analyses show that ADCP is a pivotal biologic process in pancreatic carcinogenesis. Next, CALB2, NLGN2, NCAPG and SERTAD2 are identified through multivariate Cox regression. These 4 genes are confirmed with significant prognostic value in PDAC. Then, a risk score formula is constructed and tested in PDAC samples. Finally, the correlation between these 4 genes and M2 macrophage polarization was screened. Some pivotal differentially expressed ADCP-related genes and biologic processes, four pivotal subgroup was among identified in the protein-protein network, and hub genes was found in these sub group. Then, an ADCP-related formula was set: CALB2* 0.355526 + NLGN2* -0.86862 + NCAPG* 0.932348 + SERTAD2* 1.153568. Additionally, the significant correlation between M2 macrophage-infiltration and the expression of each genes in PDAC samples was identified. Finally, the somatic mutation landscape and sensitive chemotherapy drug between high risk group and low risk group was explored. This study provides a potential prognostic signature for predicting prognosis of PDAC patients and molecular insights of ADCP in PDAC, and the formula focusing on the prognosis of PDAC can be effective. These findings will contribute to the precision medicine of pancreatic ductal adenocarcinoma treatment.

## INTRODUCTION

Pancreatic ductal adenocarcinoma (PDAC) is a life-threaten disease with lowest survival rates among major cancers and its mortality rate per years is increasing from 9^th^ to 7^th^ [1]. Positive results of computed tomography (CT) often only occurs on terminal PDAC patients, with a delayed diagnosis and poor prognosis of patients [2]. Additionally, the poor prognosis of PDAC patients is also due to high recurrence rate and early distant metastasis [3]. Aiming to prompt diagnosis and treatment, some advanced effect procedures have been put forward, including nucleic acid in circulating cancer cells, long non-coding RNA in extracellular vesicle, and some pivotal clinical characteristics [4–6]. Besides these, some individualized diagnostic methods based on sequencing and specific biological functions needs to be identified.

Among the different anti-tumor immune responds, antibody-based tumor therapy is an origin component. Specifically, there are three pivotal mechanisms in antibody-based tumor therapy, including antibody-dependent cellular cytotoxicity (ADCC), antibody-dependent cell phagocytosis (ADCP) and complement-dependent cytotoxicity (CDC) [7].

ADCP immunological therapies are described as the novel engine in precise treatment, because malignant cells could be precisely destroyed by the directly binding of antibodies and the viability for macrophage-depended phagocytosis, which is effective in the treatment of most tumors [8]. Some antibodies have been filtered and proved having directly effect on ADCP with favorable therapeutic outcome [8]. An EGFR- targeting IgG antibody called Cetuximab, has been discovered it can increase the efficacy of gemcitabine therapy and radiotherapy in pancreatic cancer [9, 10]. Since then, plenty of clinical trials were developed about the potential safety and effectiveness for cetuximab in the treatment of pancreatic cancer, most of the clinical trials showed positive results [11, 12].

The mechanisms by which cancer cells evade phagocytosis are not fully understood. Recently, Roarke A. Kamber et al. developed a platform and identified some genes that impede antibody-dependent cellular phagocytosis (ADCP). Besides CD47 and other known factors in cancer cells, the authors also found many ADCP regulatory factors by the complementary genome-wide CRISPR knockout overexpression screening platform. The author found that these regulatory factors are directly related to ADCP and play an important role in tumor malignant phenotype [13].

In our current study, we screened the expression level of ADCP regulatory factors identified by the above study from Roarke A. Kamber et al. in TCGA pancreatic cancer datasets and identified differently expressed genes (DEGs) with their potential functional pathways and hub genes above them. Then, through the combination of survival data in TCGA database and statistical analysis with cox proportional hazards regression model, a cluster of ADCP-related genes were identified with an ADCP-related risk formula in pancreatic cancer. The diagnostic and prognostic value of each screened genes and the risk formula were identified by survival analysis and receiver operating characteristic curve. Then, these findings were validated in related GEO datasets and clinical samples from PDAC patients. Additionally, the correlation between cancer-related macrophage and the screened genes was identified. Finally, somatic mutation landscape and sensitive chemotherapy drug between high risk group and low risk group was explored. Our findings reveal some pivotal ADCP-related genes in the development of PDAC and their impact on prognosis of PDAC patients. Additionally, these findings indicate ADCP-related risk formula could monitor ADCP and predict clinical outcomes in PDAC patients and lead to further research on precision therapy.

## RESULTS

### Screening differential expression ARGs in GTEx and TCGA-PAAD sequencing data

**Figure 1:**
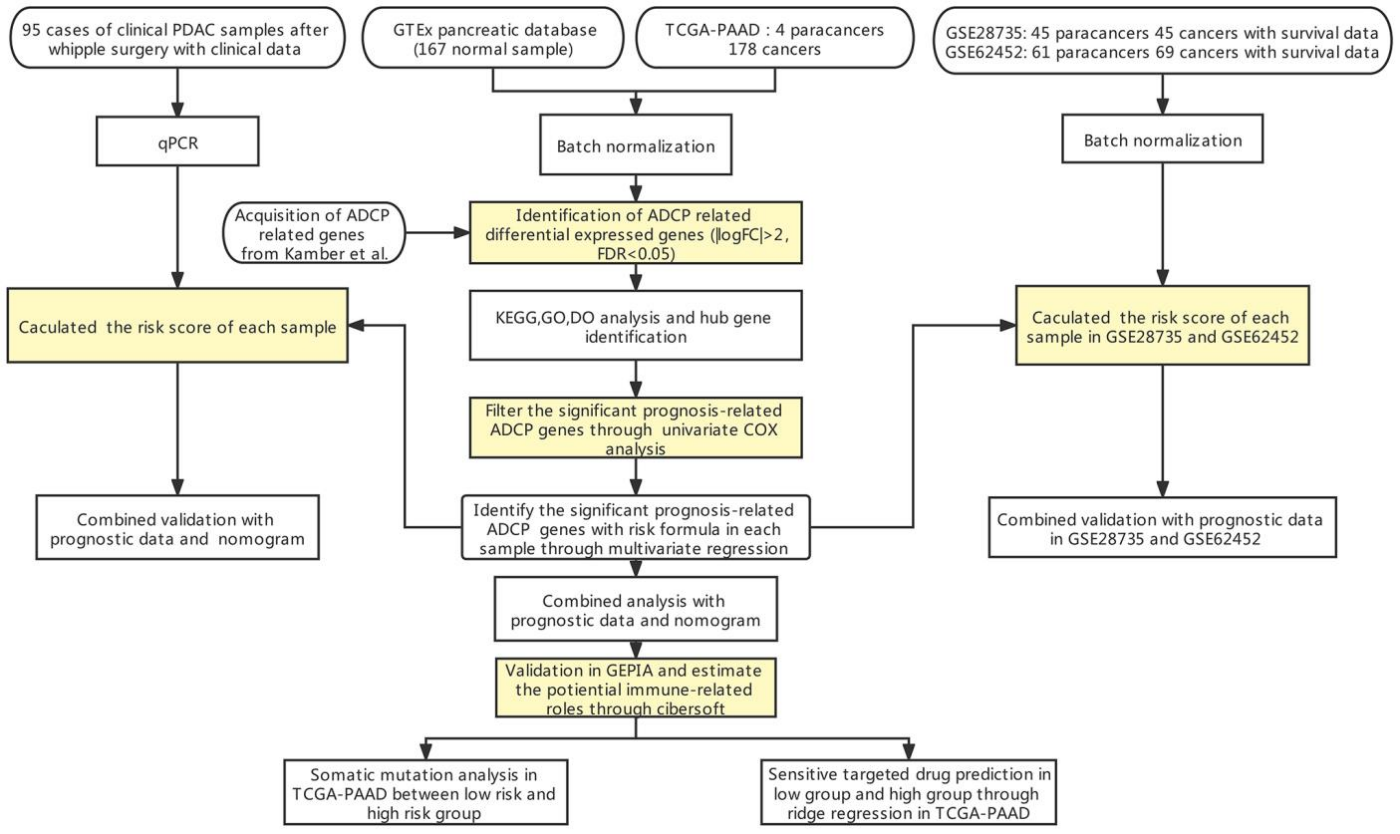
Flow chart of our study.

**Figure 2:**
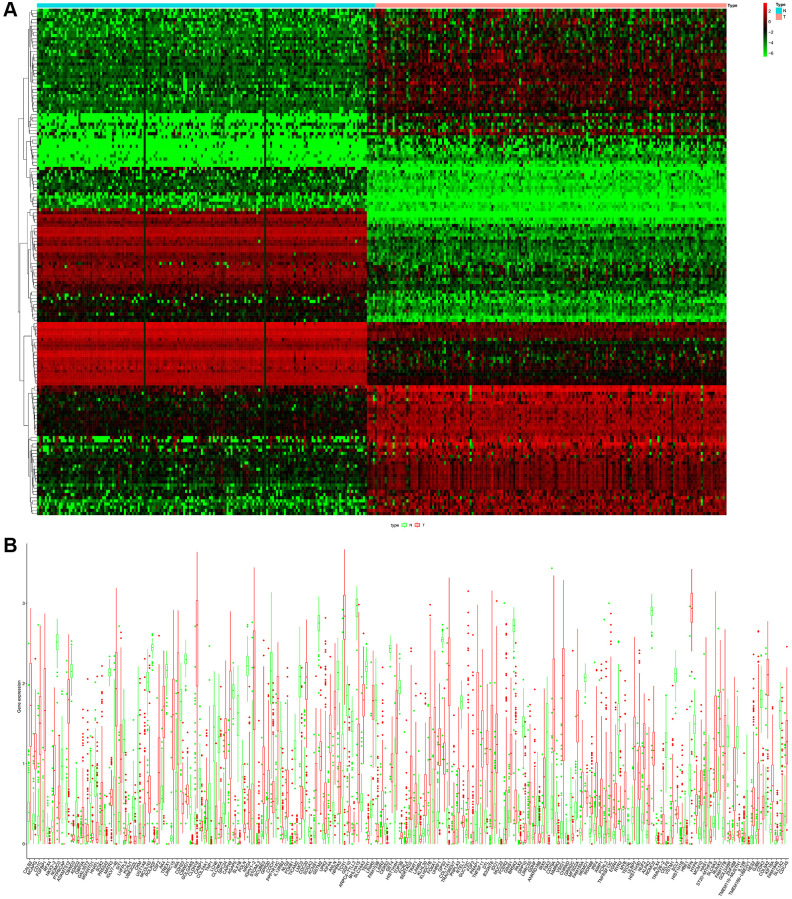
(**A**) Heatmap of the differential ARGs in the combination of GTEx data and TCGA-PAAD data. (**B**) Barplot of each differential ARGs between normal samples (green) and tumor samples (red).

The flow chart of our study has been illustrated in Figure 1. Among the expression of ARGs in GTEx and TCGA database, logFC > 2 with adjusted *p* value < 0.05 was defined as the differential ARGs. As shown in Figure 2, 160 genes were identified as the differential ARGs. The details of these genes are shown in Supplementary Table 1.

### Identification of significant pathway and hub genes

**Figure 3:**
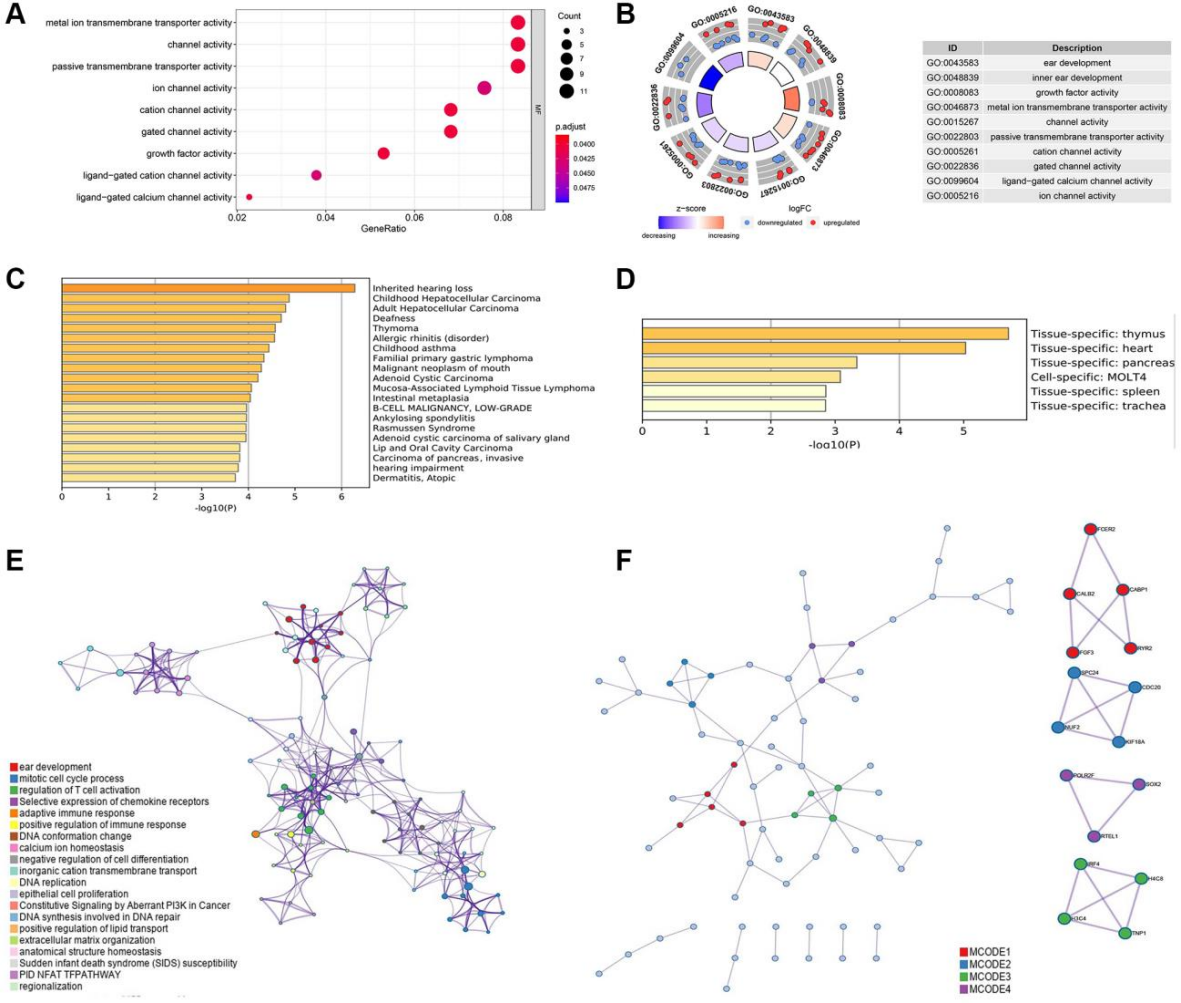
(**A**) Gene ontology (GO) analysis of the differential ARGs using R software. (**B**) Kyoto Encyclopedia of Genes and Genomes (KEGG) analysis of the differential ARGs using R software. (**C**) Disease specific analysis of the differential ARGs through Metascape online tool. (**D**) Tissue specific enrichment analysis through Metascape online tool. (**E**) Protein-protein interaction analysis with significant biologic signaling pathway through Metascape. (**F**) Hub subgroup of the whole interaction network with hub genes.

Then, based on the identified 160 differential ARGs, enriched GO pathway analysis was executed with clusterprofile package in R software and enriched GO, disease specific and tissue specific analysis is executed through Metascape (Figure 3). Some pivotal cellular ion channel is significantly enriched in screened ARGs, including metal ion transmembrane transporter activity, passive transmembrane transporter activity, ligand−gated calcium channel activity and ion channel activity (Figure 3A and 3B). In addition, disease specific analysis shows a wide range of cancer related disease was enrolled in these ARGs, including hepatocellular carcinoma, malignant neoplasm of mouth, and carcinoma of pancreas, invasive (Figure 3C). The tissue specific analysis also showed these screened ARGs significantly participate in pancreatic biologic process (Figure 3D).

The interaction among these ARGs with significant enriched pathway was shown in (Figure 3E), and several pathways were related to tumor immunity, including mitotic cell cycle process, regulation of T cell activation, adaptive immune response, and positive regulation of immune response. (Figure 3F) shows four pivotal subgroup was identified in the protein-protein network, and hub genes was found in these sub group, including FCER2, CABP1, CALB2, FGF3, RYR2, SPC24, CDC20, NUF2, KIF18A, POLR2F, SOX2, RTEL1, IRF4, H4C8, H3C4, TNP1.

### Identification of prognosis-related ARGs

**Figure 4:**
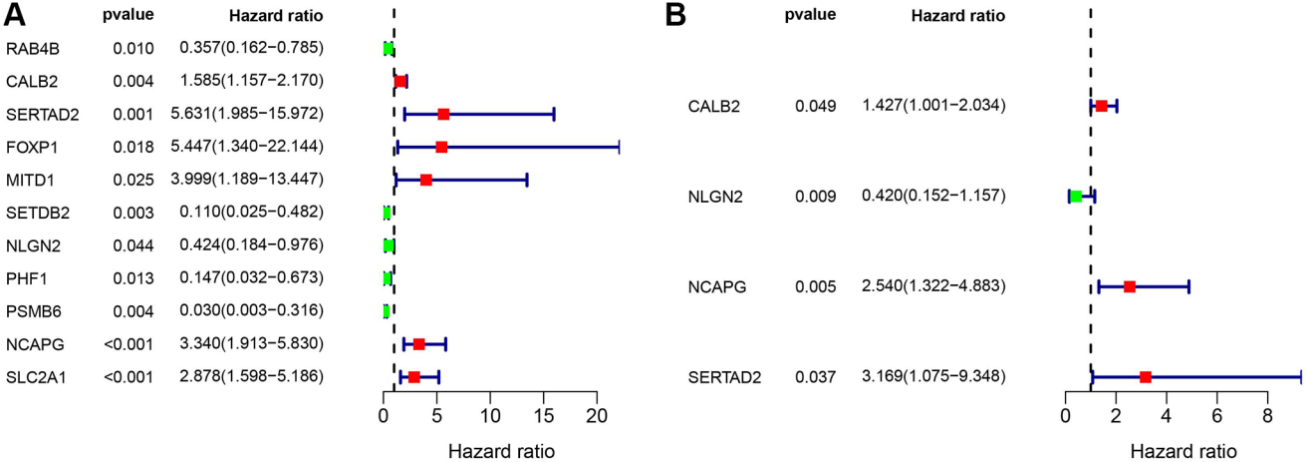
(**A**) Univariate cox regression of the ARGs in TCGA-PAAD cohort. (**B**) Multivariate cox regression of the ARGs in TCGA-PAAD cohort.

The relevance among the mRNA level of ARGs and clinical outcomes were calculated through univariate cox regression (*P* < 0.05). 11 prognosis-related ARGs were identified in TCGA-PAAD cohort (Figure 4A). Then, the 11 screened prognosis-related ARGs was analyzed through multivariate cox regression, and CALB2, NLGN2, NCAPG and SERTAD2 was identified as the significant prognosis-related genes in TCGA-PAAD cohort (Figure 4B).

### Construction and validation of the risk formula based on screened prognosis-related ARGs

Combining the multivariate analysis and the coefficient ratio of the 4 screened ARGs (Supplementary Table 2), a risk formula was constructed to estimate the risk of PDAC patients: CALB2* 0.355526 + NLGN2* -0.86862 + NCAPG* 0.932348 + SERTAD2* 1.153568.

**Figure 5:**
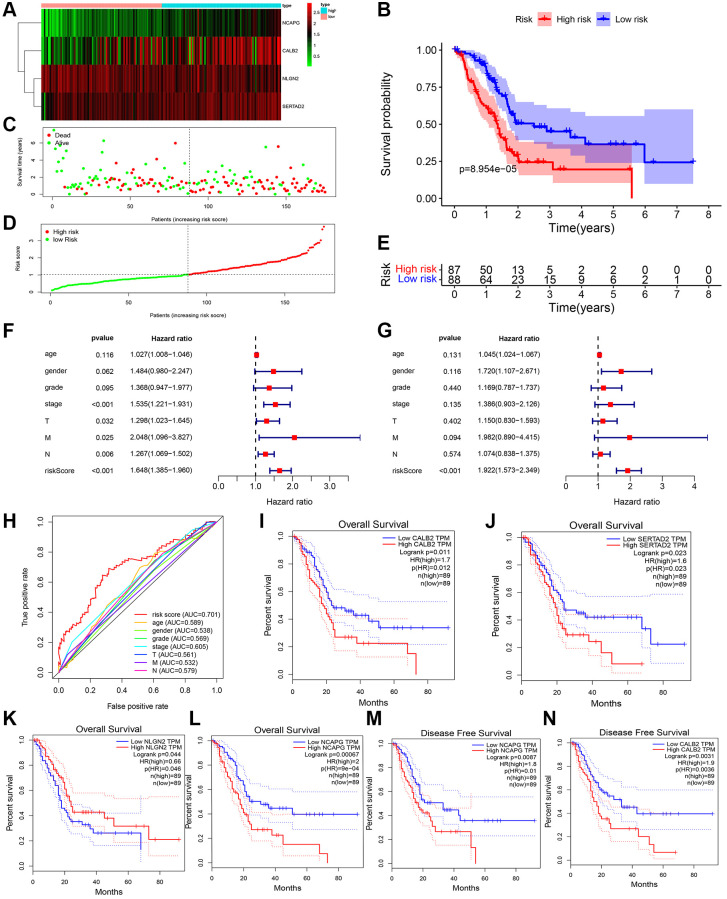
**ADCP-associated risk score of PDAC patients and validation in TCGA cohorts.** (**A**) Heatmap of the 4 screened ARGs in TCGA-PAAD cohort. (**B**) Survival analysis of high-risk group and low-risk group. (**C**) Number of patients in low risk group and high risk group. (**D** and **E**) The distribution of patients by risk score in TCGA-PAAD. (**F**) Univariate cox regression of clinical feature and risk score in TCGA-PAAD. (**G**) Multivariate cox regression of clinical feature and risk score in TCGA-PAAD. (**H**) ROC of risk score in TCGA-PAAD (**I**–**N**) Overall survival analysis and disease free survival analysis of the 4 genes in risk formula in TCGA-PAAD.

The expression of CALB2, NLGN2, NCAPG and SERTAD2 was performed in Figure 5A. The risk score was calculated, and then high-risk patients and low-risk patients were divided by median expression (Figure 5C and 5D). The prognosis of high risk patients is significantly poorer than low risk patients in TCGA-PAAD cohort (Figure 5B and 5E). Additionally, combining with the clinical features in TCGA-PAAD, the prognostic value of risk score was examined by univariate cox and multivariate cox regression (Figure 5F and 5G). Diagnostic value of the risk formula was identified in TCGA-PAAD through ROC analysis (Figure 5H). All of the 4 ARGs in the formula, including CALB2, NLGN2, NCAPG, SERTAD2, are identified with significant prognostic value in overall survival time of TCGA-PAAD patients (Figure 5I–5L). Among them, CALB2 and NCAPG were found having significant prognostic value in disease free time of TCGA-PAAD patients. (Figure 5M and 5N).

### Validation of the risk formula based on screened prognosis-related ARGs *in silico* and clinical samples

**Figure 6:**
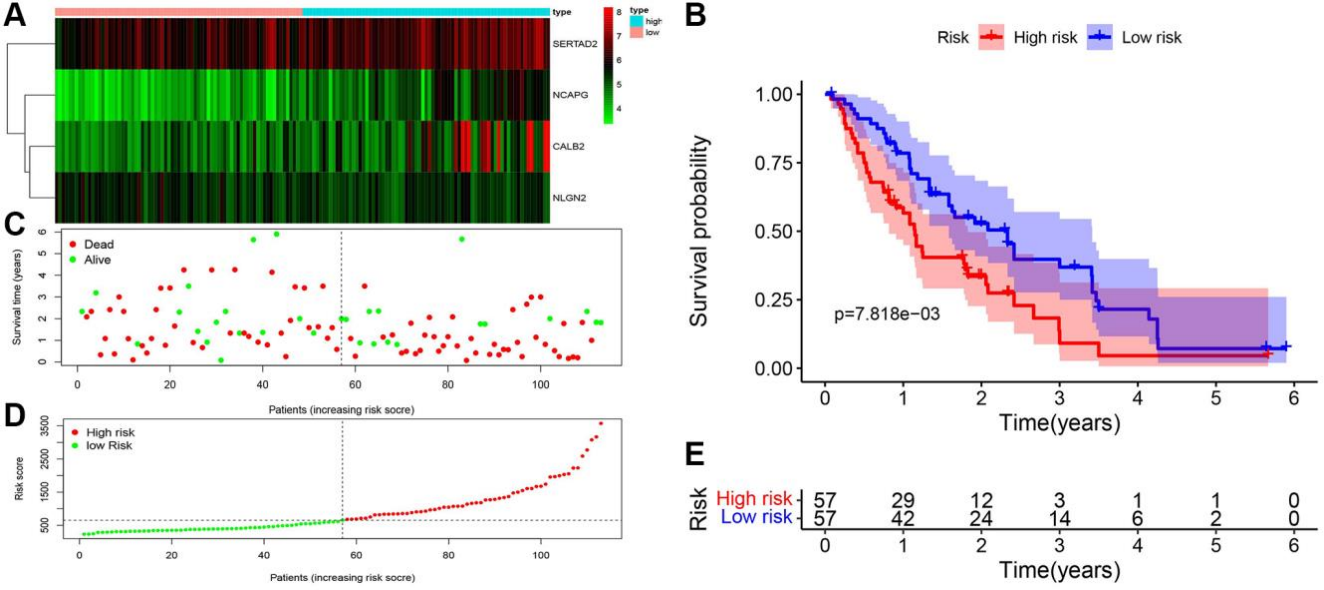
**Validation of ADCP-associated risk score in GEO cohorts.** (**A**) Heatmap of the 4 screened ARGs in GSE28735 and GSE62452. (**B**) Survival analysis of high-risk group and low-risk group. (**C**) Number of patients in low risk group and high risk group. (**D** and **E**) The distribution of patients by risk score in GSE28735 and GSE62452.

Then, the risk formula was validated in GEO database with two datasets (GSE28735 and GSE62452) after batch normalization. The risk score of 114 tumor samples in this cohort was calculated and identified with significant prognostic value (Figure 6). Specifically, the expression of CALB2, NLGN2, NCAPG and SERTAD2 was performed in Figure 6A. The risk score was calculated, and then high risk patients and low risk patients were divided by median expression (Figure 6C and 6D). The prognosis of high risk patients are significantly poorer than low risk patients in TCGA-PAAD cohort (Figure 6B and 6E).

**Figure 7:**
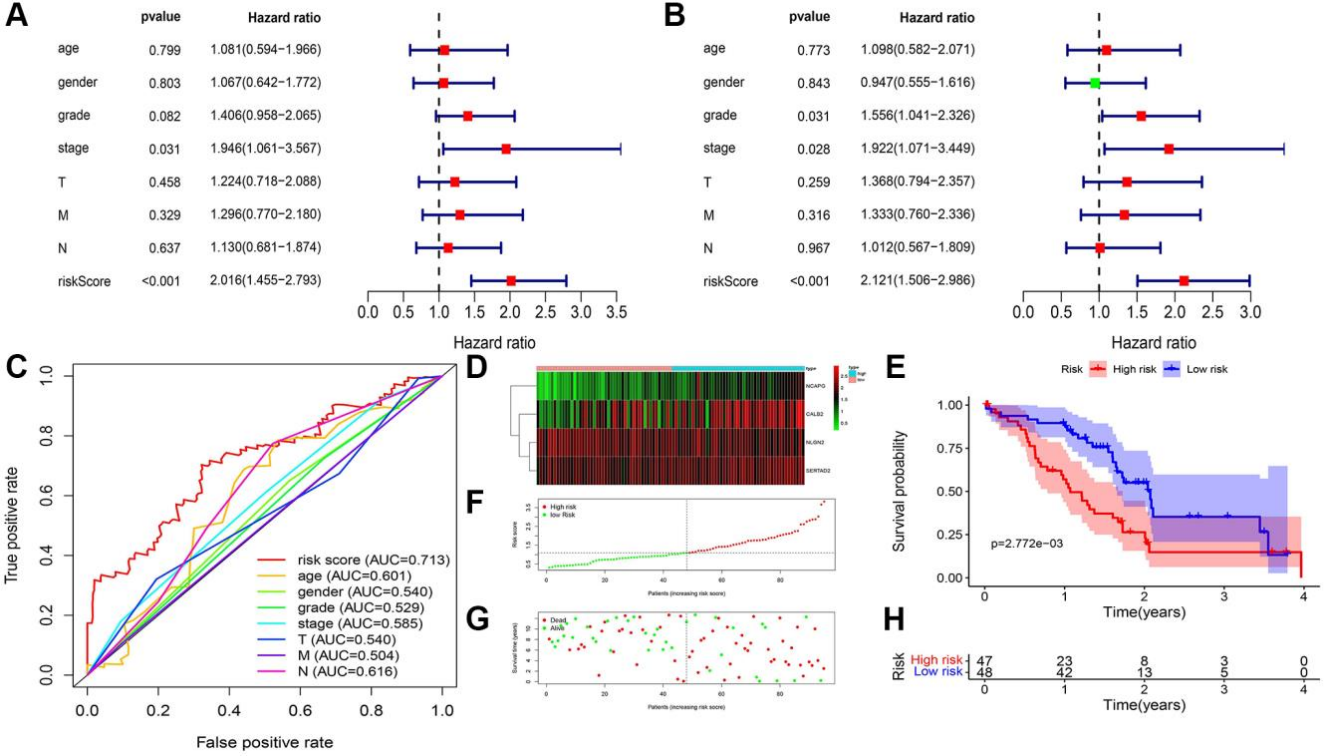
**ADCP-associated risk score of PDAC patients and validation in our local cohorts.** (**A**) Univariate cox regression of clinical feature and risk score in TCGA-PAAD. (**B**) Multivariate cox regression of clinical feature and risk score in TCGA-PAAD. (**C**) ROC of risk score in our local cohort (**D**) Heatmap of the 4 screened ARGs in TCGA-PAAD cohort. (**E**) Survival analysis of high-risk group and low-risk group. (**F**) Number of patients in low risk group and high risk group. (**G** and **H**) The distribution of patients by risk score in TCGA-PAAD.

To execute further test for the risk formula in PDAC, the mRNA expression of CALB2, NLGN2, NCAPG and SERTAD2 in 95 tissues was detected by RT-PCR and risk score was calculated through the risk formula. The baseline of patients is shown in Table 1. Combining with the clinical features of 95 clinical tumor samples, the prognostic value of risk score was examined by univariate cox and multivariate cox regression (Figure 7A and 7B). Diagnostic value of the risk formula was identified in the 95 clinical tumor samples through ROC analysis (Figure 7C). The risk score of these 95 clinical tumor samples was calculated and identified with significant prognostic value (Figure 7D–7H).

**Table 1:** Detail clinical data of qRT-PCR data from 95 samples.

	**High risk (*N* = 47)**	**Low risk (*N* = 48)**	***P* value**	**ALL (*N* = 95)**
Age > 65:			0.57	
Yes	28 (59.5%)	25 (52.1%)		53 (55.8%)
No	19 (40.5%)	23 (47.9%)		35 (44.2%)
Sex:			0.82	
Male	24 (51.1%)	20 (41.7%)		44 (46.3%)
Female	23 (48.9%)	28 (58.3%)		51 (53.7%)
Stage:			0.02	
I	8 (17.0%)	27 (56.3%)		35 (36.8%)
II	16 (34.0%)	13 (27.1%)		29 (30.5%)
III	16 (34.0%)	6 (12.5%)		22 (23.2%)
IV	7 (15.0%)	2 (4.1%)		9 (9.5%)
Grade			0.03	
G1	13 (27.7%)	18 (37.5%)		31 (32.6%)
G2	14 (29.8%)	28 (58.3%)		42 (44.2%)
G3	15(31.9%)	2 (4.2%)		17 (17.9%)
G4	5(10.6%)	0 (0%)		5 (5.3%)
T			0.454	
T1	21 (44.7%)	21 (43.8%)		42 (44.2%)
T2	14 (29.8%)	17 (35.4%)		31 (32.6%)
T3	10 (21.3%)	8 (16.7%)		18 (18.9%)
T4	2 (4.2%)	2 (4.2%)		4 (4.3%)
M			0.698	
M1	12(25.5%)	10 (20.8%)		22 (23.2%)
M0	35 (74.5%)	38 (79.2%)		73 (76.8%)
N			0.831	
N1	12 (25.5%)	14 (29.2%)		26 (27.4%)
N0	35 (74.5%)	34 (70.8%)		69 (72.6%)

### Construction of nomogram with risk score in TCGA-PAAD cohort and our local cohort

**Figure 8:**
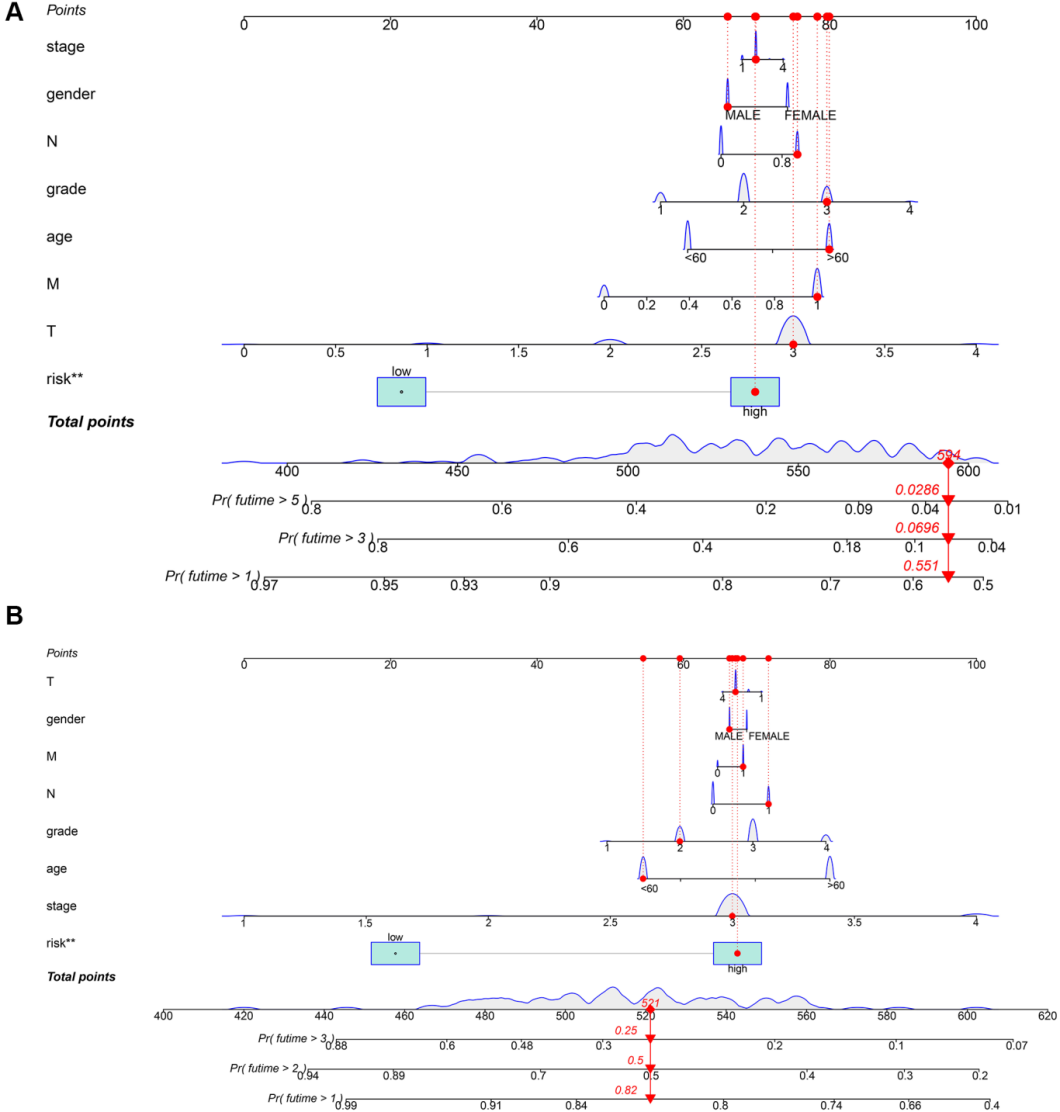
**Construction of a nomogram for evaluating prognosis.** (**A**) Nomogram for predicting the 1-, 3-, and 5 years OS of PDAC patients in TCGA. (**B**) Nomogram for predicting the 1-, 2-, and 3 years OS of PDAC patients in our local samples.

We used clinical characteristic and risk score in TCGA-PAAD (Figure 8A) and our local clinical samples to further construct nomograms (Figures 8B). The nomogram was used to assess the 1-, 3-, 5 years or 1-, 2-, 3- years survival rates of a single patient. The two nomograms indicated the risk formula is reliable, which could facilitate the clinical managements of PDAC.

### Exploring the correlation between the screened ADCP-related genes and immune phenotype

**Figure 9:**
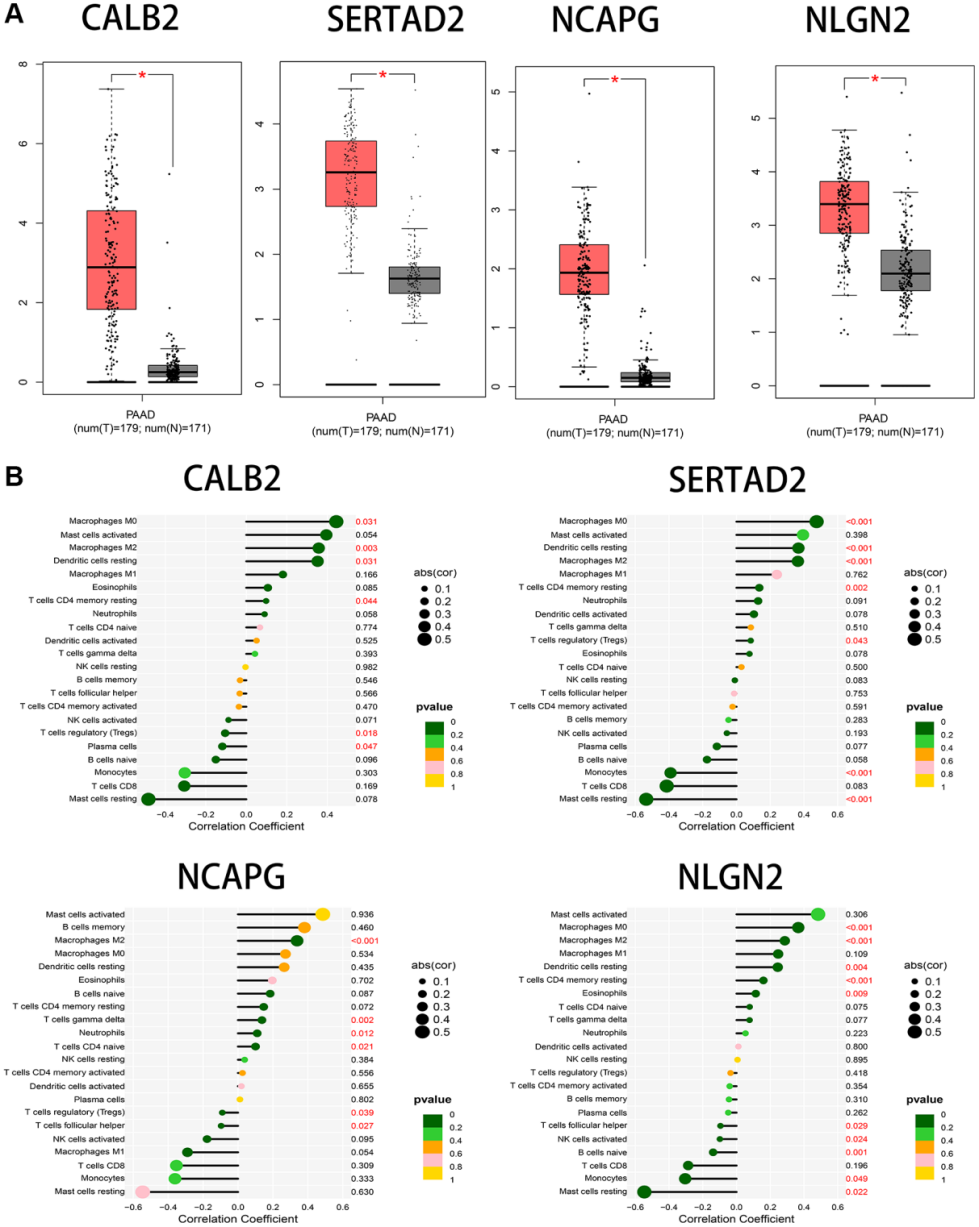
(**A**) The significant expression between tumor samples and normal samples in GEPIA database. (**B**) The correlation between immune cells infiltration and the 4 screened ARGs through cibersoft methods.

Then, the significant differential expression of the four screened genes between normal and tumor tissues was verified in PDAC patients through GEPIA database (Figure 9A). The significant correlation between each genes and exact main type of immune cells was calculated through cibersoft methods. CALB2, SERTAD2, NCAPG, NLGN2 was found only having significant positive correlation with M2 macrophages consistently (Figure 9B).

### Identify the somatic mutation landscape and predict sensitive target drugs between high risk group and low risk group in PDAC

**Figure 10:**
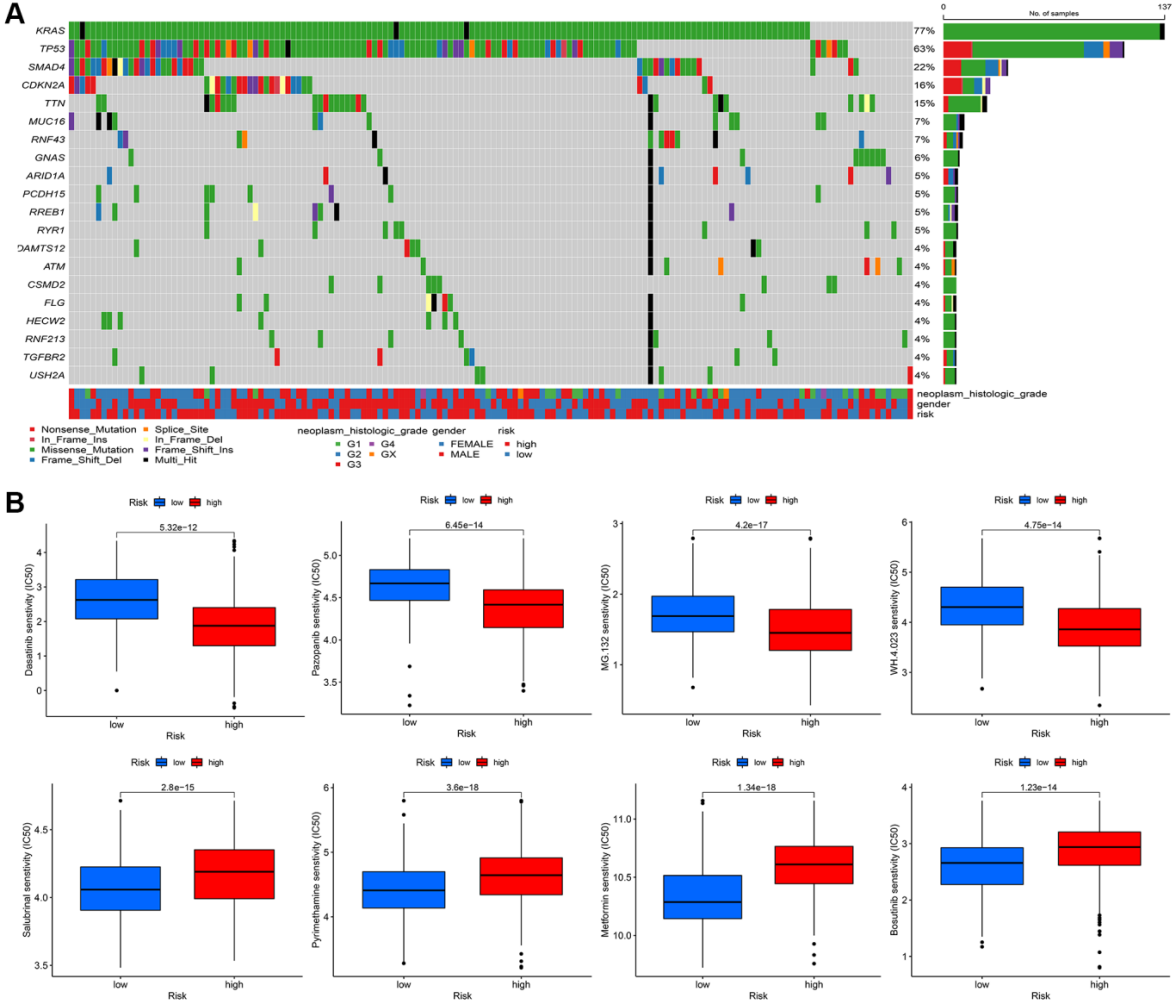
(**A**) The mutational landscape of two immune subtypes (high risk and low risk) (**B**) The potential sensitive targeted drugs in high risk group and low risk group.

Finally, we explore the somatic mutation landscape between high risk group and low risk group in TCGA-PAAD. More mutation sites and genes are observed in high risk group comparing to low risk group (Figure 10A). Dasatinib, Pazopanib, MG.132, WH.4.023 are sensitive to high risk group and Salubrinal, Pyrimethamine, Metformin, and Bosutinib are more sensitive to low risk group than high group, according to the expression data from TCGA-PAAD (*P* < 0.000001) (Figure 10B).

## DISCUSSION

As a fatal malignant tumor, PDAC causes huge social healthy burden and patients could neither be directly diagnosis in the early stage, nor be predicted the clinical outcome accurately. In this point, the prognostic marker and further basic research is in great need for the treatment of PDAC patients [14]. Among the different anti-tumor immune responds, antibody-based tumor therapy is an origin component. Specifically, there are three pivotal mechanisms in antibody-based tumor therapy, including antibody-dependent cellular cytotoxicity (ADCC), antibody-dependent cell phagocytosis (ADCP) and complement-dependent cytotoxicity (CDC) [7].

ADCP immunological therapies are described as the novel engine in precise treatment, because malignant cells could be precisely destroyed by the directly binding of antibodies and the viability for macrophage-depended phagocytosis, which is effective in the treatment of most tumors [8]. Some antibodies have been filtered and proved having directly effect on ADCP with favorable therapeutic outcome [8]. An EGFR- targeting IgG antibody called Cetuximab, has been discovered it can increase the efficacy of gemcitabine therapy and radiotherapy in pancreatic cancer [9, 10]. Since then, plenty of clinical trials were developed about the potential safety and effectiveness for cetuximab in the treatment of pancreatic cancer, most of the clinical trials showed positive results [11, 12].

The mechanisms by which cancer cells evade phagocytosis are not fully understood. Recently, Roarke A. Kamber et al. developed a platform and identified some genes that impede antibody-dependent cellular phagocytosis (ADCP). Besides CD47 and other known factors in cancer cells, the authors also found many ADCP regulatory factors by the complementary genome-wide CRISPR knockout overexpression screening platform. The author found that these regulatory factors are directly related to ADCP and play an important role in tumor malignant phenotype [13]. In this research, we used bioinformatical and statistical methods to screen the ADCP-related biomarkers with great prognostic sensitivity, based on the screened ADCP-related genes in the research from Roarke A. Kamber et al. We found FCER2, CABP1, CALB2, FGF3, RYR2, SPC24, CDC20, NUF2, KIF18A, POLR2F, SOX2, RTEL1, IRF4, H4C8, H3C4, TNP1 could be pivotal genes in the modulation of ADCP in PDAC and some pivotal cellular ion channel is significantly enriched in screened ARGs, including metal ion transmembrane transporter activity, passive transmembrane transporter activity, ligand−gated calcium channel activity and ion channel activity. To the best of our knowledge, these genes are hardly been identified as the related gene in PDAC process before [15], because the ADCP-process is a special immune-related biological process which has not been fully clarified in PDAC.

After univariate and multivariate cox regression combined with clinical outcomes, CALB2, NLGN2, NCAPG and SERTAD2 was identified as the significant prognosis-related genes. CALB2 is a calcium binding protein, has been identified playing an important role in regulating the response of colorectal cancer to 5-Fluorouracil [16]. Additionally, CALB2 is currently considered as the most sensitive and specific marker for the diagnosis of malignant mesothelioma. The mechanisms of CALB2 in malignant mesothelioma is through the binding of septin 7 on CALB2 promoter [17]. NLGN2 was found to act exclusively at GABA inhibitory synapses. Altered expression and mutations in NLGN2 and several of its interacting partners are linked to cognitive and psychiatric disorders, including schizophrenia, autism, and anxiety. In our research, NLGN2 was found to have a minimally protective impact on pancreatic ADPC-related carcinogenesis. However, high level of NLGN2 has also been found having a significant positive role for PDAC development in our current study. Unclear mechanism about NLGN2 in PDAC carcinogenesis need be further explored. NCAPG has been found as a stimulative for cardia adenocarcinoma [18], endometrial cancer [19], lung cancer [20]. Furthermore, high expression of NCAPG are relevant to poor prognosis in ovarian cancer [21] and hepatic cancer [22]. In our research, NLGN2 was found to be positive associated with PDAC development, and may play an important role in ADPC-related biologic process. In our current search, SERTAD2 was identified as a significant prognosis-related biomarker with the largest risk coefficient ratio. Previous study identified SERTAD2 as a proto-oncogene and supports the potential for SERTAD2 as a novel prognostic marker and a chemotherapeutic drug target in human cancer [23]. However, the mechanism of SERTAD2 in cancer development has not be fully elucidated. In our research, we identified SERTAD2 could promote PDAC through ADCP-related biological process with significant prognosis value. Then, the significant correlation between M2 macrophage-infiltration and the expression of each genes in PDAC samples was identified. Finally, several different chemotherapy drugs were screened as potential sensitive drugs for high risk group and low risk group. Further basic research needs to be done to verify the ADCP-related mechanism of each of the four genes and their roles in recruiting macrophages and transdifferentiation of the macrophages to M2 phenotype. However, there are some limitations in our study. First, the ADCP-related genes and risk formula we identified are based on genes expression in tumor tissue rather than in blood samples. This could weaken the diagnostic value of our risk formula because of the poor accessibility of these samples. Second, machine learning related algorithms are not been used in our study. This weakness of our current study would be overcome in our further researches.

## CONCLUSION

In conclusion, through the combination of ADCP-related genes and PDAC sequencing data, an ADCP-related formula with four genes was identified and validated in our clinical samples. The for genes identified by this formula are significant related to M2 polarization of macrophages in PDAC tumor. Different chemotherapy drugs are identified with sensitivity between high risk and low risk group.

## MATERIALS AND METHODS

### Data source

The gene expression in normal pancreas are downloaded in GTEx portal (https://www.gtexportal.org/home/index.html, 167 normal pancreas samples). Additionally, gene expression and somatic mutation data in tumor samples with paired clinical data are downloaded in The Cancer Genome Atlas (TCGA) database (https://www.cancer.gov/about-nci/organization/ccg/research/structural-genomics/tcga) named TCGA-PAAD (TCGA PAAD: 4 paracancers and 178 cancers with survival data) and two datasets in Gene Expression Omnibus (GEO) database (http://www.ncbi.nlm.nih.gov/geo/), GSE28735 and GSE62452. (GSE28735 [24]: 45 paracancers and 45 cancers with survival data. GSE62452 [25]: 61 paracancers and 69 cancers with survival data.) GSE28735 is a cohort of gene chip data collected from patients with PDAC at the University of Medicine, Göttingen, Germany. GSE62452 is another gene chip data collected at the University of Maryland Medical System at Baltimore (Baltimore, MD, USA).

### Batch normalization

Batch effect is part of the measurement results, because of the different experimental conditions. The purpose of correcting batch effect is to reduce the irrelevant differences between batches, and to identify the differences between different biological states. To remove the impact of batch effect in TCGA and GTEx samples, and GEO datasets (GSE28735 and GSE62452), SVA package was used in R software [26].

### Data acquisition

The ADCP-related gene (ARGs) list was acquired in the study from Roarke A. Kamber et al. [13]. In this study, a list of ADCP genes was screened by developed a platform for unbiased identification of factors that block ADCP using complementary genome-wide CRISPR knockout overexpression screening in cancer cells and macrophages. The ADCP genes with *P* < 0.05 was defined as ARGs and was selected to be analyzed in our current research (Supplementary Table 3). High-throughput sequencing mRNA expression data of ARGs and corresponding clinical data of Pancreatic Adenocarcinoma (PAAD) cohort were obtain from the UCSC Xena datasets (https://xenabrowser.net/datapages/).

### Differentially expressed ARGs and enrichment analysis

Differential ADCP-related genes (ARGs) expression data in TCGA-PAAD were screened by the limma package in R software (FDR <0.05, |logFC| >2). Pathway enrichment and hub gene identifications in which these 160 differential ARGs were identified with clusterprofile package [27] in R software and Metascape online tool (https://metascape.org/) [28].

### Univariate, multivariate COX regression

After 160 differential ARGs was acquired, univariate and multivariate COX regression was exerted in these ARGs using Survival and Survminer package. Specifically, univariate COX regression was used in these 160 ARGs firstly, significant ARGs was filtered and then these filtered ARGs was analyzed through multivariate COX regression (*P* < 0.05). Combined with clinical data of TCGA-PAAD samples and our local samples, including TMN staging, age, sex, risk and statues, univariate analysis was exerted to identify the significance between prognosis and clinical status.

### Survival analysis and receiver operating characteristic curve

Based on our identified risk formula, the high risk and low risk of the clinical samples from TCGA and our local clinical samples was divided. Kaplan-Meier survival analysis was exerted through Survival package between high risk group and low risk group. Combined with clinical data of TCGA-PAAD samples and our local samples, including TMN staging, age, sex, receiver operating characteristic (ROC) curve was analyzed by pROC package.

### Online database manipulation

GEPIA (Gene Expression Profiling Interactive Analysis) [29] is another database containing TCGA PDAC tumor sequencing data and GTEx normal tissue sequencing data, was used to identify the prognostic value of CALB2, NLGN2, NCAPG and SERTAD2 through Kaplan-Meier plots in optimal cut-off value and the expression of these genes among pancreatic tumor tissue and common tissue through box plots.

### Clinical samples collection

A total of 95 frozen primary PDAC samples were collected at the Department of General Surgery of First affiliated hospital of Zhengzhou University from Jan 2012 to October 2019 from PDAC patients undergoing Whipple surgery. The average follow-up time of each patients is 2.3 year. Consent was acquired from all patients in written format. This study was executed according to Declaration of Helsinki, and the Ethics Committee of First affiliated Hospital of Zhengzhou University. The baseline characteristics of PDAC patients are listed in Table 1.

### Quantitative real time polymerase

The 95 PDAC samples was isolated with TRIzol reagent (Noweizan, China) and reverse-transcribed using the HiScript II Reverse Transcriptase Kit (Noweizan, China). Real-time PCR was performed using SYBR Green (Noweizan, China). Quantitation was performed in triplicate. 2ΔΔCT method was used to calculate the expression and GAPDH was used to be as internal reference. The primers for the mRNAs are CALB2 (forward) 5′-GCAGAGCTGGCGCAGATC-3′, CALB2 (reverse) 5′-GCTCATCGTACGGCCGG TTCG-3′; NLGN2 (forward)5′-ccaaagtgggctgtgacc-3′ NLGN2 (reverse) 5′-ccaaaggcaatgtggtagc-3′; NCAPG (forward): 5′-AAGTTAGACGGGCAGTGTTATC-3; NCAPG (reverse): 5′-CAGCTTTCTGACAGCCTCTT-3; SERTAD2 (forward)5′-ATATATGTTGGGT AAAGGAGGAA-3' SERTAD2 (reverse): 5′-TGG CGC TGT AAGGTGTAAGAC-3′; GAPDH (forward) 5′-ACAGTCAGCCGCATCTTCTT-3′ and GAPDH (reverse) 5′-GACAAGCTTCCCGTTCTCAG-3′.

### Construction and validation of nomogram

Combined with clinical data of TCGA-PAAD samples and our local samples, including TMN staging, age, sex, risk and statues, two nomogram was constructed through regplot package in R software, respectively (*P* < 0.05).

### Calculation of tumor microenvironment cell infiltration

CIBERSORT were applied to quantify the relative proportions of infiltrating immune cells [30]. Spearman’s rank correlation analysis was exerted when exploring the relationship between the expression of CALB2, NLGN2, NCAPG and SERTAD2 and the immune infiltrated cells.

### Perform the somatic mutation land scape and prediction of response to chemotherapy between high risk group and low risk group

The maftools R package was utilized to analyze somatic mutation data from TCGA-PAAD and visualize the mutation waterfall plots [31]. The risk statues are also showed in the plot. All statistical values were tested by two-sided test, and *p* < 0.05 was considered statistically significant. The R package of pRRophetic was used to predict IC50 of common chemotherapeutic agents [32]. IC50 indicates the effectiveness of a substance in inhibiting specific biological or biochemical functions. The difference between high risk and low risk groups was tested by Wilcox and log-rank test (*P* < 0.000001).

### Data availability

The PAAD dataset was downloaded from TCGA database (https://tcga-data.nci.nih.gov/tcga/) and the GSE28735 and GSE62452 was downloaded from GEO database (http://www.ncbi.nlm.nih.gov/geo/). The basic code used in this study was deposited in https://github.com/inevitable48/Aging-us/.

## Supplementary Materials

Supplementary Table 1

Supplementary Table 2

Supplementary Table 3
