# Supplementary material for: Establishing and validating an ADCP-related prognostic signature in pancreatic ductal adenocarcinoma
Source: Aging (Albany NY). 2022 Aug 12;14(15):6299–315. doi: 10.18632/aging.204221 (PMC9417234; doi:10.18632/aging.204221)
Supplement: Supplementary Table 2 [file aging-14-204221-s002.pdf]

SUPPLEMENTARY TABLES

Supplementary Table 2. The result of multivariate analysis in PDAC.

ID	Coefficient	Hazard Ratio	HR.95L	HR.95H	*P* value
CALB2	0.355526	1.426931	1.001137	2.033819	0.04927
NLGN2	−0.86862	0.41953	0.152186	1.156515	0.009317
NCAPG	0.932348	2.540468	1.321665	4.88322	0.005166
SERTAD2	1.153568	3.16948	1.074573	9.34846	0.036592
